# Targeting Asymptomatic Malaria Infections: Active Surveillance in Control and Elimination

**DOI:** 10.1371/journal.pmed.1001467

**Published:** 2013-06-18

**Authors:** Hugh J. W. Sturrock, Michelle S. Hsiang, Justin M. Cohen, David L. Smith, Bryan Greenhouse, Teun Bousema, Roly D. Gosling

**Affiliations:** 1 Malaria Elimination Initiative, Global Health Group, University of California, San Francisco, United States of America; 2 Department of Pediatrics, University of California, San Francisco, United States of America; 3 Clinton Health Access Initiative, Boston, MA, United States of America; 4 Department of Epidemiology, Johns Hopkins Bloomberg School of Public Health, Baltimore, United States of America; 5 Department of Medicine, University of California, San Francisco, United States of America; 6 Department of Immunology and Infection, London School of Hygiene and Tropical Medicine, London, United Kingdom; 7 Department of Medical Microbiology, Radboud University Nijmegen Medical Centre, Nijmegen, The Netherlands.

## Abstract

Hugh Sturrock and colleagues discuss the role of active case detection in low malaria transmission settings. They argue that the evidence for its effectiveness is sparse and that targeted mass drug administration should be evaluated as an alternative or addition to active case detection.

*Please see later in the article for the Editors' Summary*

**Summary Points**

- Active case detection (ACD) is a recommended intervention in low malaria transmission settings, yet evidence for its effectiveness is sparse.
- The potential of ACD to impact transmission is hampered by the ability of current field diagnostics to detect very low density infections and continued importation of parasites, as well as the operational challenges of achieving high coverage.
- The type of ACD employed should be guided by transmission setting and an understanding of risk factors.
- Standardized monitoring and evaluation of ACD strategies should be an integral component of ACD campaigns.
- In light of the current sensitivity of field diagnostic tests, targeted mass drug administration should be evaluated as an alternative or addition to ACD in low transmission settings.

## Background

The scale-up of interventions has reduced malaria burden and transmission across a number of countries [1]–[3]. As transmission declines, it often becomes increasingly focal [4], and programs need to adapt and target the remaining parasite reservoirs, deploying resources with increasing granularity. At very low transmission intensity, elimination of malaria may involve finding and treating individual infections.

**Figure 1:**
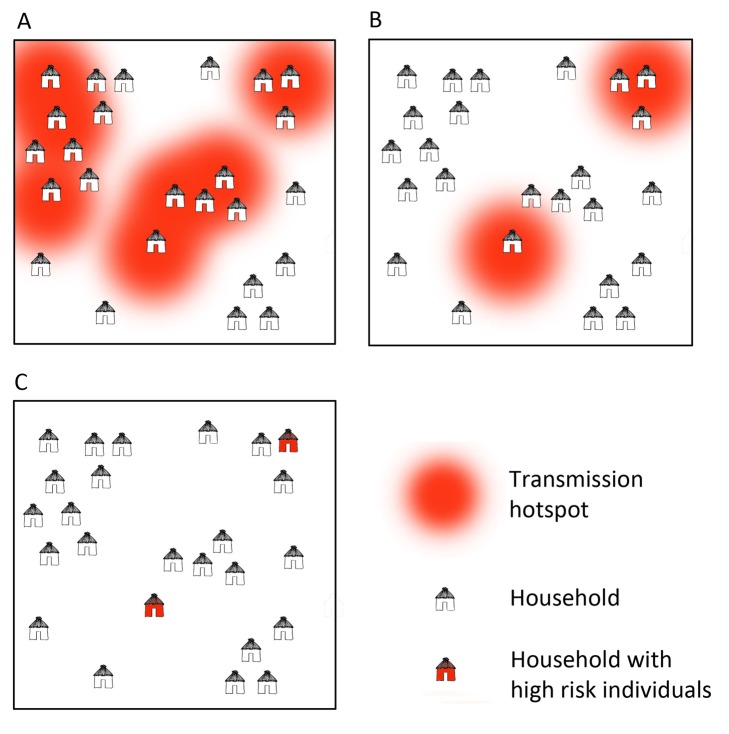
Microepidemiology of malaria in villages of varying transmission setting. In moderate/high transmission settings (A), hotspots coalesce to form a more homogeneous pattern. In lower transmission settings (B), risk becomes increasingly spatially discrete, with single households or small groups of households experiencing higher exposure. In very low transmission settings (C), risk shifts to individual households or, where transmission is occurring outside the house/village, to individuals.

**Figure 2:**
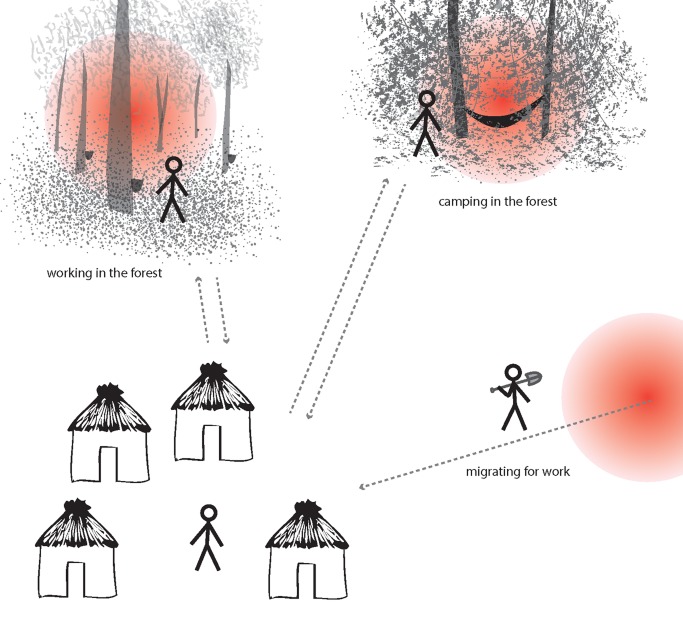
Illustration of hotpops (hot populations). While infection may be detected in individuals at their home, they acquire their infections elsewhere. For example, individuals may be exposed to infectious mosquitoes when working in particular forests overnight (e.g., rubber tappers); when camping in the forest due to occupation (e.g., loggers, miners, and military personnel); or in their place of origin (migrant laborers). These demographic groups are at high risk of infection and can seed malaria transmission to others in receptive areas.

At large spatial scales, infections tend to cluster into foci related to environmental, climatic, and ecological suitability for vectors and transmission [5]. At smaller scales within these foci, “hotspots", which consist of a household or groups of households, maintain higher transmission of malaria and a consistent reservoir of parasites throughout the year [4],[6]–[8] (Figure 1, Box 1). Infections are also clustered in certain demographic “hot" populations, or “hotpops", associated with demographic risk factors for transmission [9]–[11] (Figure 2, Box 1). In low transmission or elimination settings, strategies for detecting and targeting these clusters of infection, whether geographic or demographic, become important strategies to reduce the local parasite reservoir and interrupt transmission [12].

**Box 1. Hotspots and Hotpops**

**Hotspots:** Geographically discrete household or group of households that maintain malaria transmission throughout the year at significantly higher rates than their surroundings.

**Hotpops:** Demographically discrete groups (populations) that maintain malaria transmission at higher rates than the surrounding population.

Both hotspots and hotpops seed transmission to the surrounding populations in receptive areas. Targeting these groups prevents malaria spreading more widely.

All malaria control programs have passive surveillance systems that, to greater or lesser degrees, identify, treat, and report individuals with malaria who present to health facilities. While useful for intelligence gathering, passive surveillance alone has a limited impact on malaria transmission as only symptomatic patients receive treatment when they seek medical care. It is well known, however, that whether transmission is low or high, the majority of infections, including carriers of gametocytes (the life parasite stage responsible for onward transmission to mosquitoes), are asymptomatic [13]–[18].

To overcome the inherent limitations of passive surveillance and to target the asymptomatic parasite pool, as well as symptomatic infections in individuals who do not or cannot seek treatment, a number of programs have adopted active case detection (ACD) strategies [12]. Despite its increasing popularity across a number of countries, and recommendation by the World Health Organization (WHO) for use in malaria elimination [12], the diversity of ACD methods and the relative strengths and weaknesses of the various approaches are poorly described. In this *Policy Forum*, we discuss the potential role of ACD in malaria control and elimination. While we focus on *P. falciparum*, the discussion also includes the potential role of ACD in the control and elimination of *P. vivax*.

## Active Case Detection Methods

ACD for malaria infection has a variety of definitions and designs [19]. The WHO recently revised the definition of ACD [12] (Box 2) to differentiate methods that test only febrile individuals (fever screening) from those that target all individuals (active infection detection, aggressive active case detection, or mass screen and treat) [14],[20],[21]. While requiring more resources, approaches that target all people at risk of infection enable the targeting of the asymptomatic parasite pool. For the remainder of this discussion we use the term ACD to refer to the active detection of malaria infections in both symptomatically and asymptomatically infected individuals.

**Box 2. WHO Definition of Active Case Detection**

‘The detection by health workers of malaria infections at community and household level in population groups that are considered to be at high risk. Active case detection can be conducted as fever screening followed by parasitological examination of all febrile patients or as parasitological examination of the target population without prior screening.’ [12]

### Reactive Case Detection

Active case detection can be split into two broad types: reactive (RACD) and proactive (PACD) case detection (Table 1) [22]. RACD takes advantage of the fact that parasite carriage tends to be spatially and temporally clustered. Therefore, infections are found at higher prevalence in households in close proximity to passively detected cases [23].

**Table 1:** Characteristics of Reactive and Proactive Case Detection.

Characteristic	Reactive	Proactive
Definition	Screening and treatment for household members and neighbors of a passively detected index case	Screening and treatment in communities and among specific high risk groups without the trigger of a passively detected index case
Advantages	Allows screening to be targeted in space and timeParticipation more likely as subjects more willing to participate when index case is known to them	Able to target screening to high-risk groupsAble to support identification of asymptomatic hotspotsAble to target populations with poor access to healthcare
Disadvantages	Requires team on-call year round unless employed seasonallyMay miss populations with low or no access to health care	Community campaigns largeParticipation may be limited due to perception of low risk in low transmission sites
Recommendations for epidemiological/impact evaluation	Compare routine clinical incidence of locally acquired cases in implementation and control areas	Compare routine clinical incidence of locally acquired cases (no travel within 4 weeks) in implementation and control areas (low transmission)
	Compare infection prevalence within different radii around each index case to help inform optimal screening radius	Compare change in infection prevalence between implementation and control areas using sequential cross-sectional surveys with sensitive molecular methods (moderate transmission)
Recommendations for operational/process evaluation	Cost of implementation, proportion of cases recorded and investigated within 7 days of index case presentation, proportion of individuals screened within screening radius, mean person/time required to: (a) screen one individual; (b) identify one infection.	Cost of implementation, proportion of individuals screened, mean person/time required to: (a) screen one individual; (b) identify one infection.
	Compare performance of diagnostic test against molecular-based gold standard	
Challenges and research priorities	• Impact on transmission unknown	
	• Optimal target population not established	
	• Optimal timing and frequency not established	
	• Development of a highly sensitive and convenient diagnostic method	
	• Coverage of screening needed to affect transmission not known	
	• Methods to gain access to hard-to-reach populations required	
	• Usefulness for *P. vivax* unclear	
	• Cost-effectiveness studies required	

Despite the widespread use of RACD, no standardized guidelines have been established on either the appropriate thresholds to trigger its use or the screening radius to use. A recent compilation of national policies of countries participating in the Asia Pacific Malaria Elimination Network (APMEN), where *P. vivax* is the predominant malaria species, showed that programs employ a wide range of different RACD approaches [24]. While RACD is an intuitively appealing approach to identify infections, we know of no studies that assess its impact on transmission.

### Proactive Case Detection

PACD, which involves screening of high-risk populations, has been used extensively to reduce transmission in countries such as Taiwan [25], China [26] and Brazil [14], targeting all subjects or febrile individuals only [27] (as was common during the era of the Global Malaria Eradication Program in the 1960s [28],[29]). Field and modeling studies suggest that PACD can reduce transmission when diagnostic tests detect most infections, with the longest period of reduction occurring in lower prevalence settings [21],[30],[31]. A recent study in Burkina Faso found no impact of PACD on parasite prevalence or incidence of clinical episodes after 12 months of follow up [32]. The low sensitivity of rapid diagnostic tests (RDT) to detect all parasitaemic and gametocytaemic individuals was given as a possible explanation for the limited impact on parasite prevalence.

## Considerations for Successful ACD Implementation

Given the limited evidence for the effectiveness of ACD, the number of different options available, and the contrasting epidemiology of malaria between settings, it is highly unlikely that policy recommendations for one setting apply to any or all others. If, however, programs decide that ACD fits within the country's strategic plan, they need to consider several issues in order to maximize the potential impact of ACD.

### Choice of Method

Choosing when and what method of ACD to implement is critical (Figure 2) [33]. Neither PACD nor RACD are likely to be successful if targeted to areas with highly transient populations. In very low transmission settings, such as pre-elimination, elimination, and prevention of reintroduction stages, imported parasites are or will become the main infection source initiating chains of malaria transmission. Border screening is one PACD approach to implement to identify these infections [34]–[36]; however, it is costly, labor-intensive, and misses subjects crossing at unofficial borders. This strategy may not be practical and financially feasible in larger or less well-resourced countries, particularly those with high volumes of cross-border movement. Alternatively, individuals can be targeted by identifying the networks to which they are affiliated. For example, if an infection is thought to be imported, fellow travelers should be identified, using snowball and time-location sampling [37], and then screened and treated where appropriate.

RACD should only be conducted in receptive areas where there is potential for transmission around the residence of the index case. In these areas, RACD should take place regardless of a case being reported as imported or locally transmitted, as imported cases may also lead to local transmission. RACD is typically best suited to lower endemic settings because of the high costs involved of tracing each case. Where resources are scarce, limiting RACD to certain high-risk situations, such as within known foci or in areas with low coverage of indoor residual spraying (IRS) and/or insecticide-treated nets (ITN), could help to streamline operations and lower costs. Similarly, index households and immediate neighbors of passively detected cases should be prioritized. In very low endemic situations where the risk of malaria may not be related to place of domicile but rather is related to population characteristics such as occupation, RACD can be employed demographically rather than geographically, reactively screening networks of individuals with common risk factors.

PACD is best suited to moderate transmission settings where risk is defined in either space or time, such as areas of well-known seasonality. In such settings, campaigns should be conducted during the dry season, when mosquito densities are lowest and infections are most clustered, as this timing is likely to have the greatest impact on transmission [4],[21],[30].

### Targeting

ACD can be guided spatially by risk maps based on parasite prevalence [5], but these maps become less reliable in very low transmission settings as prevalence approaches 0% [38],[39]. Maps or models based on passive surveillance data may help to delineate transmission zones [40]. Where the location of hotspots and profile of hotpops appear to be stable over time [6], initial rounds of PACD can inform targeted future rounds of PACD and other interventions.

A better understanding of risk factors would allow ACD to be focused on those individuals at highest risk. Risk factors can be identified through analysis of routine data; however, risk assessments are more accurate when made using case-control study methods [41], such as are frequently used for outbreak investigations [42].

### Coverage of Population

Coverage (the proportion of the target population tested during ACD) is unlikely to be perfect, and is influenced by the accuracy of the maps used to guide operations, the availability of resources, and the mobility and willingness of populations to be tested. To maximize coverage, programs should screen communities at times when people are at home, record who is missed, and return to improve coverage where possible. Establishing the number of people missed and their demographic characteristics is important to ensure that those at highest risk of malaria infection are not missed.

Community involvement is likely to be key to achieving high coverage. In Peru, community volunteers perform weekly house-to-house visits to allow screening and treatment of confirmed infections [43]. The use of local volunteers may also improve coverage amongst individuals who are away from the home during the daytime and may be missed by conventional programmatic ACD.

### Choice of Intervention

A key component of any ACD campaign is the intervention that is implemented following the detection of a case. Treatment of *P. falciparum* with artemisinin combination therapies (ACT) alone will hinder the development of clinical malaria and can kill immature gametocytes, reducing the probability of onward transmission to mosquitoes [44]–[46], but it may not be sufficient to prevent transmission shortly after treatment [47]. Integrating a treatment drug that acts against mature gametocytes, such as an 8-aminoquinoline, has the potential to further reduce transmission [48],[49]. Recently, WHO changed its recommendation on the use of primaquine for *P. falciparum* (Box 3) [50].

**Box 3. WHO Primaquine Recommendation**

In areas threatened by artemisinin resistance where single dose primaquine as a gametocytocide for *P. falciparum* malaria is not being implemented, and elimination areas which have not yet adopted primaquine as a gametocytocide for falciparum malaria, a single 0.25 mg base/kg primaquine dose is recommended. This should be given to all patients with parasitologically-confirmed falciparum malaria on the first day of ACT treatment regardless of G6PD status, except for pregnant women and infants <1 year of age.

ACT, artemisinin combination therapy; G6PD, glucose-6-phosphate-dehydrogenase.

In addition to drug treatment, other interventions, such as targeted vector control, may improve the impact of ACD [21]. Establishing the optimal vector control method should be based on local epidemiology, because long-lasting insecticidal nets (LLIN) and IRS are unlikely to be successful where transmission occurs outside the house or is related to occupation. In such settings, larval source management, personal protective measures such as repellent, insecticide-treated clothing, and insecticide treated hammock nets should be used where appropriate [51]–[53].

### Impact and Effectiveness

Mathematical modeling studies support the idea that PACD using ACT reduces transmission. However, results from recent field studies are inconclusive, with Sutcliffe et al. showing an impact on transmission and Tiono et al. showing no impact [31],[32]. For RACD, we are not aware of any studies measuring the impact on transmission. Despite this dearth of evidence, both strategies are implemented widely. Thus, there is an urgent need to develop and install monitoring and evaluation tools, using standardized indicators to assess processes and impact (Table 1). In low-to-moderate transmission settings, such impact evaluation may be possible, as done by Sutcliffe *et al.* (2012), using sequential cross- sectional surveys to assess changes in infection prevalence. Use of sensitive molecular methods is preferable over RDT for such an evaluation, to allow detection of a larger proportion of asexual and sexual parasite stages. In elimination settings, however, such methods are inappropriate due to the paucity of positives. Evaluation must rely on other transmission metrics, such as incidence and serological responses. Randomizing campaigns to areas, or adopting a step wedge design, should be used to help control for the effect of possible confounding factors, such as climate. In addition to evaluating epidemiological indicators, programs can evaluate their operational efficiency using simple, key indicators of programmatic performance; e.g., the proportion of cases recorded and investigated within 7 days of presentation of the index case for evaluating RACD. Similarly, assuming global positioning system (GPS) coordinates are collected, the proportion of households covered for both RACD and PACD can be estimated using freely available satellite imagery [54]. Costs of campaigns should be recorded to allow assessment of cost-effectiveness to enable comparison with other interventions and to assess long-term financial feasibility.

### *P. vivax*

While there are commonalities with ACD for *P. falciparum*, ACD for *P. vivax* control and elimination faces unique challenges. Firstly, *P. vivax* infections tend to be maintained at low parasite densities [55]. Secondly, *P. falciparum*-specific RDT used in many countries are unable to detect other *Plasmodium* species [56],[57] and newer RDT that detect non-falciparum species still need evaluation in the field [58]. Thirdly, *P. vivax* (and *P. ovale*) has dormant liver stages (hypnozoites), which currently are impossible to detect. ACD for *P. vivax* may, therefore, require several rounds to capture individuals when their infections relapse, often without causing clinical symptoms. Sero-diagnosis, whereby anyone who is sero-positive is assumed to be infected and is treated [59], is one potential approach to overcome this challenge. A second approach is mass drug administration (MDA). However, treatment of the liver stage infection at present requires treatment with primaquine, an 8-aminoquinoline that can produce acute haemolytic anemia in individuals who are glucose-6-phosphate-dehydrogenase (G6PD) deficient [60]. Tafenoquine, a new 8-aminoquinoline under development, induces a similar effect; studies are ongoing to identify safe and effective dosages that demonstrate efficacy. Until such dosages are determined, administration of primaquine or tafenoquine requires initial testing for G6PD deficiency. While tests for G6PD deficiency are available, more sensitive and inexpensive point of care tests for G6PD deficiency are needed [61].

## Screening Test Sensitivity and Mass Drug Administration

RDT and microscopy are the diagnostic method of choice for ACD [23],[31],[62]. With increased use of more sensitive molecular methods, it is becoming clear that, contrary to traditional thought, the proportion of sub-patent infections (below the density detectable by microscopy and RDT) appears to increase with decreasing transmission [63],[64]. Due to residual levels of immunity, the proportion of infections that are sub-patent may also be particularly high in areas that have experienced recent declines in transmission. While patent asymptomatic infection may be responsible for the majority of transmission in many settings, because of a positive correlation between sexual parasite density and transmission to mosquitoes, sub-patent infections in very low transmission settings are estimated to make up 20 to 50% of all human-to-mosquito transmissions [64].

Detecting sub-patent infections requires sensitive molecular diagnostic methods, such as polymerase chain reaction (PCR) or loop-attenuated isothermal amplification (LAMP) [46],[63],[65]. At present, the use of PCR and LAMP for ACD is impractical because of their cost, infrastructure requirements, and long turn-around times. Although the use of molecular methods will certainly increase the proportion of the true reservoir of infections that is detectable, very low density infections may still be missed. While more sensitive field diagnostics are being developed, PCR and LAMP can be used to quality assure RDT and microscopy and to identify infections missed by other methods [66],[67].

Serology, or the detection of antimalarial antibodies, cannot be used to identify who has current infection during ACD. However, when the prevalence of infection detected by RDT or PCR is low, evidence of recent or past infection can be used to identify high-risk geographic regions or populations, or conversely to confirm absence of transmission [68]–[70]. Sero-diagnosis may also serve as a surrogate for potential liver carriage of *P. vivax* [59].

**Figure 3:**
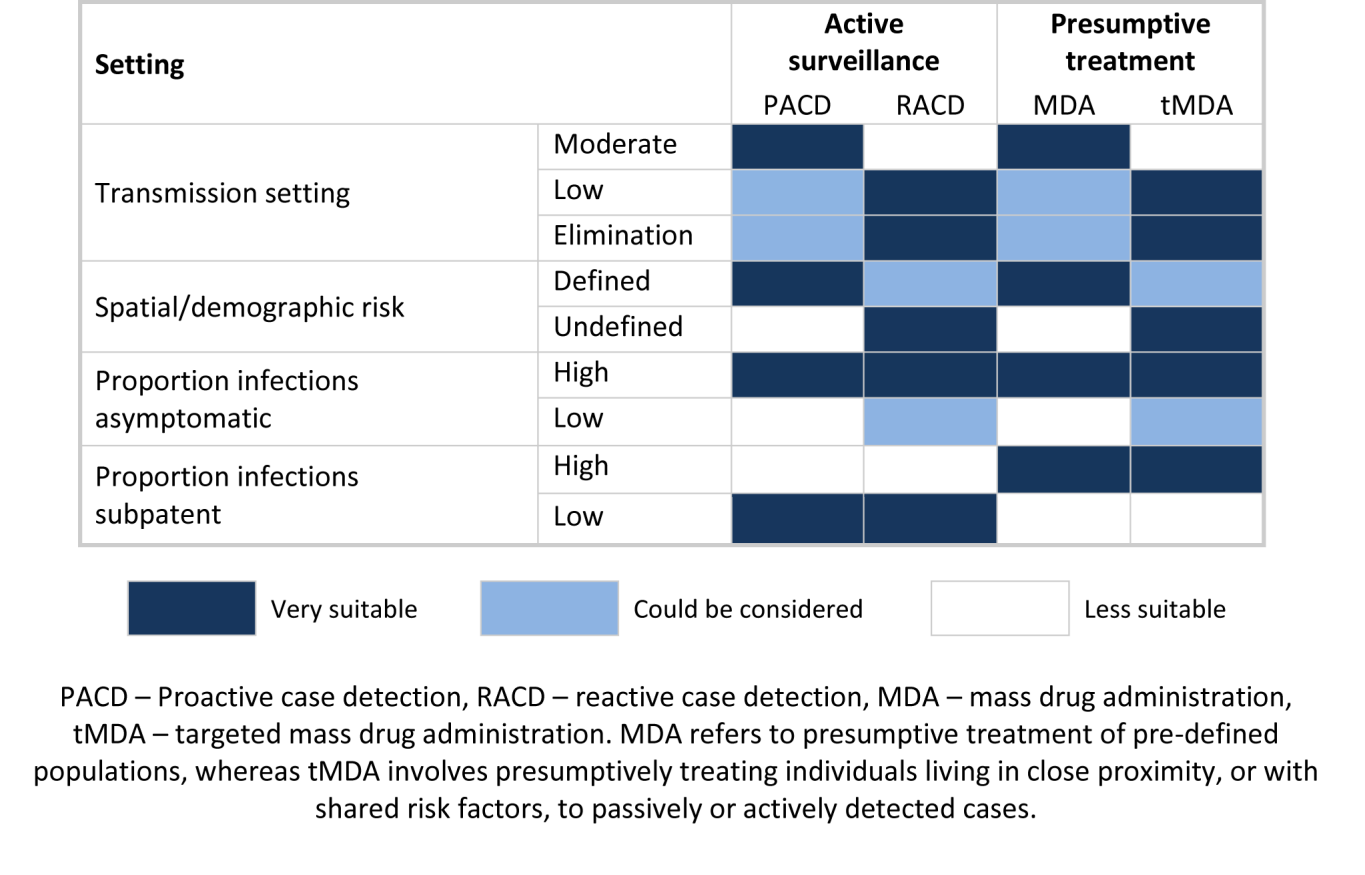
Potential application of different active surveillance and mass drug administration approaches to reduce transmission. Due to the resource requirements of tracing cases back to their home, reaction case detection (RACD) is best suited to lower transmission settings. Similarly, to avoid large amounts of unnecessary treatments, mass drug administration (MDA) is better suited to higher transmission settings; lower transmission areas may benefit from a more targeted approach. Where risk factors are well defined, proactive case detection (PACD) and MDA are good options. RACD and targeted mass drug administration (tMDA) are useful where risk factors are not well defined, as passively or actively detected cases can be used to identify at-risk populations. Where the proportion of asymptomatic infections is high, passive surveillance does not suffice and additional active surveillance and presumptive treatment are required. Where the proportion of sub-patent infections is high, active surveillance using current diagnostics is less likely to impact transmission, and presumptive treatment (MDA or tMDA) should therefore be considered.

An alternative to ACD, which overcomes the issue of missed infections, is MDA to populations with pre-defined risk factors, such as all individuals within known hotspots or migrant workers arriving from malaria endemic countries [71],[72] (Figure 3). Where risk factors are not well defined, an effective approach might be targeted MDA (tMDA) to households or groups of households identified via passively or actively detected cases. A similar household treatment approach has been suggested for schistosomiasis [73],[74]. Such an approach warrants investigation in the context of malaria control, although the correct drug combination needs to be explored. At a stage where the number of programs implementing ACD is increasing, further rigorous evaluation of ACD, and comparison with MDA with regard to effectiveness, cost-effectiveness, and operational feasibility, is critical.

## Conclusions

ACD strategies are adopted by a number of malaria control programs worldwide. Despite their popularity, the different approaches used are poorly defined and evaluated, and the factors that affect their effectiveness are not well understood. Key challenges include missing infections due to inadequately sensitive diagnostics, missing individuals due to low coverage of those most at risk, dealing with imported parasites, and diagnostic and therapeutic difficulties of non-falciparum parasites. Given these challenges, programs implementing ACD need to consider several factors. The type of ACD employed should be guided by transmission setting; RACD is better suited to low transmission settings, whereas PACD is better suited to moderate/low transmission settings. To maximize its impact, ACD should be targeted based on geographic and demographic risk. Where these risks are not well known, RACD may be a more appropriate option over PACD. Achieving high coverage should be a priority and requires operational methods, such as involving community volunteers. Once infections are identified, appropriate interventions, including drug treatment and targeted vector control, should be implemented. Finally, the development and installation of standardized tools to monitor and evaluate ACD strategies is essential to establish the cost-effectiveness of prolonged campaigns and to ensure the most efficient distribution of limited resources. More research on the relative cost-effectiveness and operational feasibility of ACD strategies as well as MDA is needed to enable the development of evidence-based guidance.

## Abbreviations

ACD: active case detection
ACT: artemisinin combination therapy
G6PD: glucose-6-phosphate-dehydrogenase
GPS: global positioning system
IRS: indoor residual spraying
ITN: insecticide-treated nets
LAMP: loop-attenuated isothermal amplification
LLIN: long-lasting insecticidal nets
MDA: mass drug administration
RACD: reactive case detection
PACD: proactive case detection
PCR: polymerase chain reaction
RDT: rapid diagnostic tests
